# Surgical strategy of laparoscopic partial nephrectomy: It is more suitable to use transperitoneal approach in anterior tumor patients and retroperitoneal approach in posterior tumor patients

**DOI:** 10.3389/fonc.2023.1115668

**Published:** 2023-02-10

**Authors:** Yijian Li, Li Huang, Wentao Liu

**Affiliations:** ^1^ Department of Urology, The Second Xiangya Hospital, Central South University, Changsha, Hunan, China; ^2^ Clinical Nursing Teaching and Research Section, The Second XiangYa Hospital of Central South University, Changsha, Hunan, China

**Keywords:** laparoscopic, partial nephrectomy, retroperitoneal, transperitoneal, renal carcinoma

## Abstract

**Background:**

Previous surgical strategy of transperitoneal laparoscopic partial nephrectomy (TLPN) and retroperitoneal laparoscopic partial nephrectomy (RLPN) for treatment of renal cell carcinoma (RCC) mainly depend on surgeons’ preference. The aim of this study was to evaluate whether performing TLPN for anterior tumors and RLPN for posterior tumors is a more beneficial strategy.

**Method:**

214 patients underwent TLPN or RLPN at our center were retrospectively collected and 1:1 matched for surgical approach, tumor complexity as well as operator. Baseline characteristics and perioperative outcomes were evaluated and compared, respectively.

**Result:**

Regardless of tumor location, RLPN was associated with a faster operative time, a quicker time to first oral intake and hospital discharge compared to TLPN approach while other baseline and perioperative outcomes were comparable between groups. After taking tumor location into consideration, TLPN has an advantage in operating time (109.8 *vs* 115.3 mins, p = 0.03) and ischemic time (20.3 *vs* 24.1 mins, p = 0.001) for anterior tumor, while RLPN has an advantage in operating time (103.5 *vs* 116.3 mins, p<0.001), ischemic time (21.8 *vs* 24.8 mins, p = 7 0.001) and estimated blood loss (65.5 *vs* 85.4 ml, p = 0.01) for posterior tumor.

**Conclusion:**

The selection of approach should be also dependent of the tumor location, instead of only dependent of surgeons’ experience or preference.

## Introduction

Renal cell carcinoma (RCC) has been found as the sixth most common malignancy in men and the tenth in women worldwide, leading to the death of over 140,000 patients every year, with an increasing incidence year by year (1).

Partial nephrectomy (PN) has been extensively used to treat the stage T1 renal tumors in recent years (2). Transperitoneal and retroperitoneal approach can be used for minimal invasive PN. However, current guidelines and relevant literatures do not have a decision-making strategy for selecting the transperitoneal and retroperitoneal approaches. As reported by previous meta-analysis, there has been no major difference between the perioperative and long-term outcomes of the transperitoneal and retroperitoneal approaches. It has been concluded that the selection of surgical approaches mainly depends on the personal preference of the surgeons (3). Considering differences in transperitoneal and retroperitoneal approaches, it can be inferred that each tumor has its own appropriate choice. Meanwhile, the clinical dilemma whether patients with an anterior tumor should preferably receive transperitoneal PN and, conversely, patients with a posterior tumor should preferably receive retroperitoneal PN has never been well clarified. Although previous retrospective study has relied on this issue and concluded that transperitoneal and retroperitoneal robot-assisted partial nephrectomy (RAPN) offer equivalent perioperative morbidity, and functional and pathological outcomes, regardless of tumor’s location (4). However, there are a large number of differences in operational technique and convenience between robot-assisted and laparoscopic technique (5). It is still unknown whether transperitoneal laparoscopic PN (TLPN) is more suitable for anterior tumours, and conversely, retroperitoneal laparoscopic PN (RLPN) is more suitable for posterior tumours.

To fill this gap, this study relied on a large retrospective database of TLPN and RLPN, and perform a comprehensive comparison of perioperative characteristics, morbidity and functional outcomes between TLPN and RLPN, especially focusing on subgroup analysis according to tumor location. To be specific, we assumed that TLPN offers effective outcomes in anterior tumors while RLPN offers favorable outcomes in posterior tumors and tried to establish a surgical strategy in laparoscopic PN (LPN).

## Materials and methods

### Patients

From January 2015 to December 2020, patients who received LPN at Second Xiangya Hospital of Central South University were retrospectively collected. The patients studied are those suspected of having a single cT1 renal tumor with CT or MRI. Patients with prior abdominal surgery or related retroperitoneal surgery history were excluded. The renal tumor fell into the anterior tumor group and the posterior tumor group in accordance with tumor location6. The LPN was performed using transperitoneal and retroperitoneal approaches in our hospital. The anterior tumor group and posterior tumor group receiving LPN were 1:1 matched for surgical approach, tumor complexity as well as operator. The routine follow-up of the respective patient was performed at three, six months and then annually. At each visit, serum creatinine and B ultrasound were performed.

The groups were compared in terms of baseline characteristics, perioperative outcomes with the use of means and percentages. Patient characteristics consisted of age, gender, body mass index (BMI), ASA score, tumor size, as well as tumor complexity. Tumor complexity was determined in accordance with R.E.N.A.L. score system (6). Perioperative data consisted of operative time, estimated blood loss (EBL), warm ischemic time (WIT), time to first oral intake, hospitalization time, positive margin rate, complications and postoperative eGFR change. Perioperative complications were categorized using Clavien grading system (7). Postoperative eGFR were evaluated at 6 months after surgery.

### Surgery

Five surgeons performed the surgeries and surgeons were 1:1 matched when collecting cases to minimize the bias of experience and skill of the surgeons. The surgical method for RLPN had reported in our previous study (8). Generally, three ports were applied for either TLPN or RLPN, and additional ports were made if needed. Briefly, the kidney was first dissociated as much as possible with a cap of perinephric fat remained on the surface of the suspected tumor and the artery of kidney was freed. After clamping the renal artery by the bulldog clip, the tumor was sharply excised with standard resection using laparoscopic cold scissors. The closure of the entire tumor bed was performed with two layer of barbed suture. Then, the renal artery was unclamped and the bleeding of tumor bed was carefully checked.

### Statistical analysis

The statistical analysis was conducted using SPSS 25.0 software. Perioperative data were compared through the Mann-Whitney U test (median and range of continuous variables) and Chi-square analysis (categorical variables). A P value < 0.05 had statistical significance. Propensity score matching analysis was conducted by Logistic regression.

## Results

On the whole, 469 patients who received partial nephrectomy were collected, and 214 patients were recruited in this study after propensity score matching, in which 107 had TLPN, and 107 had RLPN. To be specific, there were 110 patients with posterior tumors, in which 55 patients received TLPN, and 55 patients received RLPN. While 104 patients were anterior tumors, with 52 patients undergoing TLPN and 52 patients undergoing RLPN. Clinical characteristics of patients are presented in **Table 1**. There was no significant differences between groups in terms of age, gender distribution, BMI, ASA score, tumor size and R.E.N.A.L. score (all P>0.05).

**Table 1:** Clinical baseline characteristics of patients.

Variables	P-RLPN	P-TLPN	A-RLPN	A-TLPN	P* value
Number	55	55	52	52	
Age, year	54.5 ± 10.2	53.5 ± 9.2	50.5 ± 9.8	52.5 ± 8.9	all>0.05
Gender, n					all>0.05
Male	31	29	30	28	
Female	24	24	22	24	
BMI, kg/m2	23.1 ± 2.5	22.91 ± 2.8	23.4 ± 2.1	22.4 ± 2.9	all>0.05
ASA score, n(%)					all>0.05
1-2	44(80)	46(83.6)	45(86.5)	42(80.7)	
3-4	11(20)	9(16.4)	7(13.5)	10(19.3)	
Tumor size (cm)	2.7 ± 0.6	2.9 ± 0.8	2.9 ± 0.7	3.1 ± 0.7	all>0.05
R.E.N.A.L. score					all>0.05
Low+ Intermediate complexity	49	48	47	46	
High complexity	6	7	5	6	
Preoperative eGFR	84.8± 15.3	85.2 ± 16.1	84.9 ± 14.5	86.1 ± 16.2	all>0.05

*p value of comparing each two groups.

For overall perioperative outcomes of RLPN and TLPN patients, there were no significant differences in WIT, EBL and positive margin rate between the two groups (P >0.05). However, RLPN group achieved favorable results in terms of operating time (109.2 ± 14.1 *vs* 113.2 ± 12.7, P < 0.05), time to first oral intake (2.0 ± 1.1 *vs* 2.4 ± 1.3, P < 0.05) and postoperative hospital stay (4.3 ± 0.9 *vs* 4.7 ± 1.0, P < 0.05) comparing to TLPN group. There were 23 patients of perioperative complications (12 in RLPN *vs* 11 in TLPN, P=0.8), 14 patients were Clavein Grading 1-2 (8 in RLPN *vs* 6 in tLPN, P=0.6), and 9 patients were Clavein Grading 3-4 (4 in RLPN *vs* 5 in TLPN, P=0.7). Each group had three patients undergoing superselective embolization after surgery (**Table 2**). No significant difference was observed between RLPN and TLPN group in transfusion rate (4.7% *vs* 5.6%, P = 0.8) and eGRF at six months postoperatively.

**Table 2:** Perioperative outcomes of patients with RLPN and TLPN.

Variables	RLPN	TLPN	P value
Number	107	107	
Operating time, min	109.2 ± 14.1	113.2 ± 12.7	**0.03**
WIT, min	22.9 ± 5.3	22.6 ± 5.3	0.7
Estimated blood loss, mL	74.8 ± 48.4	82.4 ± 46.2	0.2
Positive margin, n(%)	2(1.8)	3(2.8)	0.7
Time to first oral intake, days	2.0 ± 1.1	2.4 ± 1.3	**0.02**
Postoperative hospital stay, days	4.3 ± 0.9	4.7 ± 1.0	**0.002**
Overall perioperative complications, n(%)	12(11.2)	11(10.3)	0.8
Clavien Grading 1-2, n(%)	8(7.5)	6(5.6)	0.6
Clavien Grading 3-4, n(%)	4(3.7)	5(4.7)	0.7
Transfusions, n (%)	5(4.7)	6(5.6)	0.8
Superselective embolization, n	3(2.8)	3(2.8)	1
Change in 6 month postop eGFR (mL/min/1.73 m2)	-4.3 ± 11.8	-5 ± 10.8	0.7

WIT, warm ischemic time. Statistically significant values are highlighted in bold.

The perioperative results of subgroup analysis according to tumor location were listed in **Table 3**. For posterior tumor, patients in the RLPN group had an advantage in terms of operative time (103.5 ± 12.1 *vs* 116.3 ± 13.2, P < 0.001), WIT (21.8 ± 4.9 *vs* 24.8 ± 4.8, P = 0.001) and EBL (65.5 ± 49 *vs* 85.4 ± 48.2, P = 0.01). There was no significant difference between RLPN and TLPN in terms of positive margin rate, postoperative complications and transfusion rate. To be specific, one patients in RLPN and two patients in TLPN received superselective embolization after surgery. According to the results of estimate glomerular filtration rate (eGRF) at six months postoperatively, there was no significant difference between the RLPN and TLPN groups (-4.2 ± 11.5 *vs* -5.1 ± 10.8, P =0.7). For anterior tumors, patients in the TLPN group showed an advantage of operative time (109.8 ± 11.3 *vs* 115.3 ± 13.6, P = 0.027) and WIT (20.3 ± 5.0 *vs* 24.1 ± 5.5, P = 0.001) as compared with the RPLN group. There was insignificant difference between RLPN and TLPN group in EBL, positive margin rate, postoperative complications and transfusion rate. To be specific, two patients in RLPN group and one patients in TLPN group received superselective embolization after surgery. There was no significant difference in the eGRF at six months postoperatively between the and TLPN groups (-4.4 ± 12.1 *vs* -4.9 ± 10.9, P =0.8).

**Table 3:** Perioperative outcomes of patients with RLPN and TLPN subgrouped by tumor location.

Variables	For posterior tumor			For anterior tumor		
	RLPN	TLPN	P	RLPN	TLPN	P
Number	55	55		52	52	
Operating time, min	103.5 ± 12.1	116.3 ± 13.2	**<0.001**	115.3 ± 13.6	109.8 ± 11.3	**0.027**
WIT, min	21.8 ± 4.9	24.8 ± 4.8	**0.001**	24.1 ± 5.5	20.3 ± 5.0	**0.001**
Estimated blood loss, mL	65.5 ± 49	85.4 ± 48.2	**0.01**	84.7 ± 46.2	79.2 ± 44.2	0.5
Positive margin, n(%)	1(1.8)	2(3.6)	0.6	1(1.9)	1(1.9)	0.6
Overall perioperative complications, n(%)	6(10.9)	6(10.9)	1	6(11.5)	5(9.6)	0.7
Clavien Grading 1-2, n(%)	4(7.2)	3(5.5)	0.7	4(7.7)	3(5.8)	0.7
Clavien Grading 3-4, n(%)	2(3.6)	3(5.5)	0.6	2(3.8)	2(3.8)	1
Transfusions, n (%)	2(3.6)	4(7.2)	0.4	3(5.7)	2(3.8)	0.6
Superselective embolization, n	1(1.8)	2(3.6)	0.5	2(3.8)	1(1.9)	0.6
Change in 6 month postop eGFR (mL/min/1.73 m2)	-4.2 ± 11.5	-5.1 ± 10.8	0.7	-4.4 ± 12.1	-4.9 ± 10.9	0.8

WIT, warm ischemic time. Statistically significant values are highlighted in bold.

## Discussion

As medical technology has been leaping forward, the treatment of renal cancer has gradually changed from the traditional open surgery to minimally invasive, and partial nephrectomy has also become the mainstay of therapy for early renal cancer. In comparison with the traditional open surgery, minimally invasive surgery shows the advantages of less trauma, less blood loss, shorter hospital stay, etc. (9). The approaches for partial nephrectomy primarily consist of transperitoneal and retroperitoneal approaches. Urologists will select the surgical approach in accordance with their experience and personal habits, and both approaches exhibit their advantages and disadvantages. To formulate a surgical strategy, a matching analysis based on the tumor location was conducted on patients with anterior and posterior renal tumors who received TLPN and RLPN, respectively.

Regardless of tumor location, there were only slight differences in operating time, time to first oral intake and postoperative hospital stay between TLPN and RLPN, whereas other perioperative outcomes were comparable, which was consistent with existing meta analyses (3, 10). The meta-analysis focusing on RAPN indicated that retroperitoneal RAPN was equally safe and efficacious in complication, conversion, WIT, EBL and positive surgical margin, compared with transperitoneal RAPN, except for a marginally significant advantage of shorter operation time in retroperitoneal approach (3). Likewise, the meta-analysis focusing on LPN drew a conclusion that RLPN has an advantage in operation time and length of hospital stay as compared with TLPN, while RLPN and TLPN offer equivalent perioperative morbidity and functional outcomes (10). However, the study design of existing studies did not consider the potential confounders that significantly affect treatment selection and patient outcomes, such as tumor location and complexity. Moreover, the previous meta-analysis were not applicable to postoperative renal function because of the difference in methodologies between studies. Conversely, this study performed a comparison of TLPN and RLPN according to the tumor location and taken the renal function into consideration.

In this study, we mainly focused on the subgroup analysis and tried to verify whether TLPN is more suitable for anterior tumors, and, conversely RLPN is more suitable for posterior tumors. According to the results of this study, TLPN has an advantage in operating time and WIT for anterior tumor, while RLPN has an advantage in operating time, WIT and estimated blood loss.

WIT has been the major concern of surgeons when performing partial nephrectomy. To provide a bloodless operative field improving tumor excision and renal reconstruction, transient renal hilar clamping is required during PN, thereby causing renal warm ischemia injury and even renal failure. Though the effect of WIT on renal function remains controversial, recent studies considered 25 min as the acceptable safety threshold of WIT during PN, and these studies demonstrated that every minute of hilar clamping exerts a deleterious effect on renal function outcomes (11, 12). Given the mentioned conclusion, every effort to minimize the WIT is valuable. As we expected, the surgical strategy of laparascopic partial nephrectomy by exploiting tumor location can effectively minimize the WIT. Unlike robotic PN which is more convenient, some difficulties remain for performing either TLPN or RLPN for tumors in particular anatomic location. To take the posterior tumors an examples, adequate surgical isolation and rotation of the whole kidney to make the tumors closed to the ventral to the greatest extent possible is required when the PN is performed using the transperitoneal approach. However, some difficulties remain in resection of tumor and close the parenchymal defect even after the adequate surgical isolation. In contrast, it is more convenient to complete the procedure using retroperitoneal approach. Consistent with the analysis of this study, Takagi et al. drew a conclusion that robotic PN using retroperitoneal approach have better surgical outcomes than that using intraperitoneal approach for posterior renal tumors, and they indicated that robotic PN using retroperitoneal approach is optimal for selected lateral renal tumors (13). Moreover, some renal tumors located in specific lowest pole are seems relatively difficult to performed by laparascopic retroperitoneal approaches in our normal practice, which needs to pay additional attention. Besides making decision-making based on tumor location, there are some other surgical techniques to minimize the WIT (e.g., selective arterial clamping (14), early unclamping (15), as well as off-clamp (16)), and we consider the combination of above strategies would expand the protective gains of renal function. In addition,

Before any technique being widely applied, the safety of patients is always the priority to be considered. Complications in our series in each groups were uncommon and comparable. Each group had patients undergoing transfusions or superselective embolization, whereas no patients were converted to radical nephrectomy. Another problem concerned is the protection of renal function. Though there was no difference in the change in six-month postoperative eGFR between groups, it is reasonable to consider that the shorter WIT would result in minimized injury to the kidney as we previously reported (17).

To the best of our knowledge, this study is the first time to conduct a matching analysis of TLPN and RLPN by drawing upon the location of renal tumor. The greatest significance of this study is that the choice of the approach should not be only dependent of the surgeon’s expertise as previous reported but consider other factors (e.g., tumor location). This retrospective non-randomized analysis attempted to eliminate potential confounders that might affect the reliability of the results. Patients with anterior renal tumor or posterior renal tumor RLPN were 1:1 matched to surgical approach, tumor complexity and operator to minimize the bias, and the baseline characteristics were comparable between the two groups.

In brief, for lateral renal tumors, the retroperitoneal approach showed better surgical outcomes, which consisted of shorter WIT and lower EBL as compared with the transperitoneal approach, while the transperitoneal approach led to better outcomes for anterior renal tumors regarding to WIT and EBL. The selection of approach should be also dependent of the tumor location, instead of only dependent of surgeons’ experience or preference.

## Data availability statement

The raw data supporting the conclusions of this article will be made available by the authors, without undue reservation. Requests to access the datasets should be directed to WL, xyeyylwt@csu.edu.cn.

## Ethics statement

Written informed consent was obtained from the individual(s) for the publication of any potentially identifiable images or data included in this article.

## Author contributions

All listed authors contributed to (1) conception and design, acquisition of data, and analysis and interpretation of data; (2) drafting the article or revising it critically for important intellectual content; (3) final approval of the version to be published; and (4) agreement to be accountable for all aspects of the work. All authors contributed to the article and approved the submitted version.
